# Anisotropic thermal conductive properties of cigarette filter-templated graphene/epoxy composites

**DOI:** 10.1039/c7ra11574a

**Published:** 2018-01-03

**Authors:** Zhiduo Liu, Yapeng Chen, Wen Dai, Yuming Wu, Mengjie Wang, Xiao Hou, He Li, Nan Jiang, Cheng-Te Lin, Jinhong Yu

**Affiliations:** Key Laboratory of Marine Materials and Related Technologies, Zhejiang Key Laboratory of Marine Materials and Protective Technologies, Ningbo Institute of Materials Technology & Engineering, Chinese Academy of Sciences Ningbo 315201 China Jiangnan@nimte.ac.cn linzhengde@nimte.ac.cn yujinhong@nimte.ac.cn; University of Chinese Academy of Sciences 19 A Yuquan Rd., Shijingshan District Beijing 100049 China

## Abstract

Herein, a cigarette filter-templated graphene/epoxy composite was prepared with enhanced thermal conductive properties. The through-plane thermal conductivity of the epoxy composite was up to 1.2 W mK^−1^, which was 4 times that of it in the in-plane (0.298 W mK^−1^) after only 5 filtration cycles. The thermal conductive anisotropy and improvement in the through-plane thermal conductivity of the epoxy composite were attributed to the particular structure of cigarette filter-templated graphene in the epoxy matrix. The unique structure formed effective conductive pathways in the composite to improve the thermal transportation properties. The excellent thermal transportation properties allow the epoxy composite to be used as an efficient heat dissipation material for thermal management applications.

## Abstract 2

We report a facile method to prepare cigarette filter-templated graphene/epoxy composites with excellent thermal transport performance.

## Graphical abstract

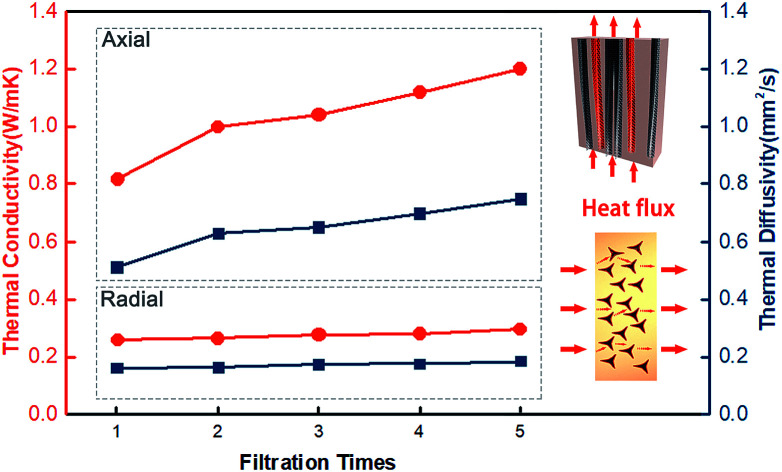

## 1. Introduction

With the development of modern electronic and electrical industries, cooling has become the main factor that affects the working stability and lifetime of devices.^1,2^ Thus, aided by the miniaturization of electronic devices, there is a growing demand for high thermal conductive materials for electronic components. Polymer materials are generally good candidate materials.^3^ Due to its excellent chemical resistance, high mechanical strength, and insulation, epoxy is considered an ideal material for electronic packing.^4^ However, its thermal conductivity is only 0.2 W mK^−1^, which enormously limits its widespread use as an electronic packing material.^5^ Therefore, multiple high thermal conductivity nanomaterials, such as boron nitride nanosheets, alumina, and graphene, have been introduced into epoxy resin to enhance its thermal transport properties.^6–11^ However, simple blending-processed epoxy-based composites commonly cannot achieve high thermal conductivity with a low nanofiller loading.^12–14^ Thus, the design and fabrication of effective heat conductive paths in the composites are crucial for the next-generation of electronic packing materials.

Graphene is considered to have the highest thermal conductivity (5300 W mK^−1^) among the materials known to date.^15^ With a two-dimensional (2D) nanostructure of sp^2^-bonded carbon atoms arranged in a honeycomb pattern, graphene has become one of the marvel carbon materials today.^16,17^ Because of its high thermal conductivity and large specific surface area, graphene is an ideal nanofiller to enhance the thermal conductivity of an epoxy matrix.^18^ When the composites were prepared through simple blending, graphene tended to aggregate and sedimentate in the epoxy matrix as its concentration increased.^19,20^ To avoid this problem, three-dimensional (3D) frameworks have been proposed to obtain a high thermal conductivity in polymer-based composites. Yang *et al.*^21^ fabricated a 3D cellulose/graphene-supported aerogel and encapsulated it with polyethylene glycol. The thermal conductivity of the composite was 1.35 W mK^−1^, which was 463% higher than that of the pristine polyethylene glycol. Chen *et al.*^22^ constructed a cellulose nanofiber-supported 3D interconnected boron nitride nanosheet aerogelskeleton *via* a sol–gel and freeze-drying process. Then, epoxy was impregnated into the 3D framework, with a 9.6 vol% boron nitride nanosheets content, and the thermal conductivity of the composite reached 3.13 W mK^−1^. However, among all the 3D structure-templated composites, few of them possessed an anisotropic structure. 3D-templated bulk composites with anisotropic thermal diffusivity have rarely been reported in the literature.

Herein, we have developed a strategy to build 3D graphene-based composites, cigarette filter-templated graphene/epoxy (CFTG/Ep) composites, with anisotropic thermal diffusivity. At first, a graphene suspension was passed through a cigarette filter (CF) to obtain cigarette filter-templated graphene (CFTG). After several filtration cycles, the as-obtained CFTG was immersed in epoxy resin to obtain the CFTG/Ep composite. The aligned CFTG can form effective heat transport pathways in the composite and enhance the thermal diffusivity at a quite low graphene loading. The incorporation of CFTG into the epoxy matrix shows a significant enhancement in the thermal diffusivity, especially in the axial direction. The thermal conductivity in the axial direction was 1.2 W mK^−1^ after only 5 filtration cycles. However, the thermal conductivity in the radial direction was only 0.298 W mK^−1^, indicating strong anisotropy. The thermal conductivity of the epoxy composite was greatly improved with a low graphene loading. Thus, the CFTG/Ep composite is promising for use as a thermal management material.

## 2. Experimental

### 2.1. Materials

Cigarette filters (CFs) were purchased from Shanghai Zhengyou Industry and Trade Co., Ltd. (China). Commercial graphene nanoplatelet (GNP) powders were produced by the Ningbo Institute of Materials Technology and Engineering, Chinese Academy of Sciences (China). Cycloaliphatic epoxy resin (6105) and methylhexahydrophthalic anhydride (MHHPA) were obtained from DOW Chemicals (U.S.A.) and Shanghai Li Yi Science & Technology Development Co. Ltd. (China), respectively. Neodymium(iii) acetylacetonate trihydrate (Nd(iii)acac) purchased from Aldrich Chemicals was used as the latent catalyst. All other chemicals were of analytical reagent grade and used without further purification.

### 2.2. Preparation of the CFTG/Ep composite

**Scheme 1:**
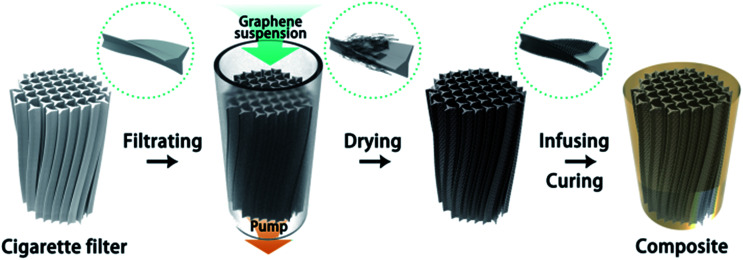
Schematic of the fabrication process of the CFTG/epoxy composites.

A solution method was adopted to prepare the GNP dispersion. At first, the GNP powder was dispersed in ethanol by applying tip sonication for 5 min at room temperature. Then, the paper cover wrapped on the CFs was removed. The pristine CF was filled in a flexible pipe to ensure that the GNP dispersion only flowed through the CF using an injection syringe. The CF turned into black after several cycles of injection and ejection. In addition, the surface of the CF was assembled with the GNP after the drying process. A quantity of Nd(iii)acac was added to a cycloaliphatic epoxy resin and subsequently stirred at 80 °C in a three-necked flask for 2 h. The resulting homogeneous solution was mixed with its curing agent (MHHPA) at a 100 : 95 ratio. The mixture was mixed using a speed mixer at a speed of 3000 rpm for 5 min. Then, the homogeneous mixture was infused into the assembled filter and then placed into a vacuum drier to remove the air bubbles from the solution. Finally, after the curing process, the samples were naturally cooled down to the room temperature and then diced using a low speed diamond cutter for different characterizations. The preparation process of the epoxy composite with cigarette filter-templated graphene (CFTG) is illustrated in Scheme 1.

### 2.3. Characterization

The microstructures of the samples were obtained using a JEOL JEM-2100 (TEM, JEOL, Japan) instrument at an acceleration voltage of 200 kV. The optical microscopy (OM) images were obtained using an optical microscope (OM, Leica DM2500M, Germany). The fractured surface of the composites was examined using field emission scanning electron microscopy (FE-SEM, QUANTA FEG250, USA) at an acceleration voltage of 10 kV. The samples were broken, and the fractured surface was coated with a thin gold layer to avoid the accumulation of charge in SEM. X-ray photoelectron spectroscopy (XPS) was carried out *via* a Kratos AXIS Ultra DLD spectrometer using Al Kα excitation radiation (*hν*: 1253.6 eV). Raman spectroscopy was conducted employing a laser wavelength of 532 nm (RENISHAW plc, Wotton-under-Edge, UK). The thermal diffusivity of the composites was determined using an LFA 447 Nanoflash apparatus (Netzsch, Germany) at room temperature. The thermal conductivity *λ* (W mK^−1^) was calculated as a multiplication of the density (*ρ*, g cm^−3^), specific heat (*C*_p_, J g^−1^ K^−1^), and thermal diffusivity (*α*, mm^2^ s^−1^), namely, *λ* = *ρ* × *C*_p_ × *α*. The samples were prepared with a square shape, 10 mm in lateral size, and a thickness of about 1.2 mm. The IR images were obtained using an infrared camera (Fluke, Ti400, USA).

## 3. Results and discussion

### 3.1. Characterization of graphene

**Fig. 1:**
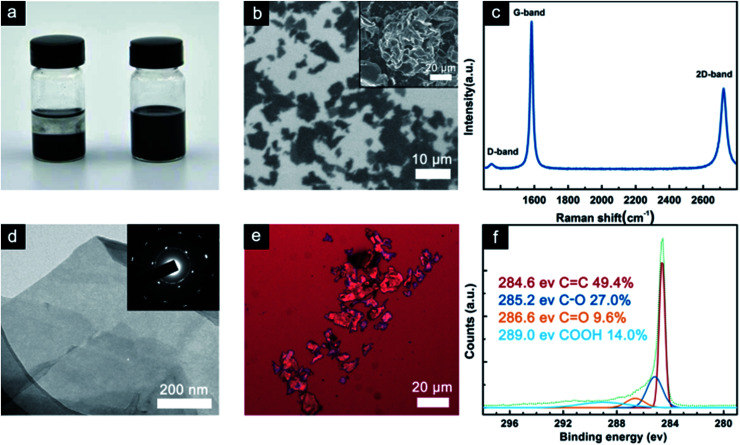
(a) An image showing the GNP dispersion before and after washing and tip sonication, (b) SEM image of the washed GNPs; the pristine GNPs powder is shown in the inset, (c) Raman spectrum of the GNPs, (d) TEM image of the GNPs; the HR-TEM image is shown in the inset of (d), (e) OM image of the GNPs, and (f) XPS C 1s spectrum of the GNPs.

Fig. 1(a) show the GNP powder dispersed in ethanol before and after washing and tip sonication. These images were obtained after 2 h of deposition at room temperature. As can be seen, the GNP suspensions before washing and tip sonication were deposited on the bottom, whereas the washed GNP suspension exhibited better colloidal stability in ethanol. In general, the dispersion of the fillers in solvent was affected by the particle–particle or sheet–sheet interactions and particle–solvent or sheet–solvent interactions.^23^ An obvious precipitation was observed in the GNP dispersion before washing and tip sonication due to the distinct agglomeration of graphene. However, the washed GNP suspension was visually observed as a homogeneous solution, indicating that its 2D plane structure and large specific surface area effectively prevented aggregation. The SEM image of the washed GNPs powder is shown in Fig. 1(b). The GNPs have a flaky sheet structure with a width of about 10 μm and below.

The inset of Fig. 1(b) shows the pristine GNP powder displaying the aggregation of graphene. Raman spectroscopy can directly reflect the thickness and quality of the graphene sheets. The Raman spectrum of the GNPs is shown in Fig. 1(c), from which we can see that the G-band at 1579 cm^−1^ corresponds to the first-order scattering of the E_2g_ mode.^24^

In addition, the D-band (1345 cm^−1^) intensity is related to disordered, sp^3^-hybrided carbon, which can be activated at the edges.^24^ The *I*_D_/*I*_G_ ratio reflects the impurities and defects in the GNPs structure, and its low value predicts a high intrinsic thermal conductivity.^25,26^ The 2D band (2708 cm^−1^) is known to be very sensitive to the graphene layers in a flake. From the position and shape of the 2D peak, we can draw a conclusion that the GNP flakes are constituted by few-layer graphene.^26^ This fact is further confirmed using TEM analysis. TEM was employed to determine the morphology and thickness of the GNPs. As shown in Fig. 1(d), a large and transparent GNP sheet with wrinkles was observed on the copper grid. The wrinkled surface was proposed to reinforce the interfacial interaction with the epoxy matrix. In addition, the inset of Fig. 1(d) shows the selected area electron diffraction (SAED) pattern, which shows that it has a multiple crystalline structure, indicating that it consists of several layers.^27^ In Fig. 1(e), the typical OM image shows the morphology of the GNP deposited on a Si substrate by dip-coating. The size and thickness of the GNPs were in agreement with the SEM and TEM results.

XPS was used to explore the chemical character of the GNPs, as shown in Fig. 1(f). It revealed the elemental composition of the GNPs in which the oxygen-containing functional groups also enabled the strong interfacial interaction between GNPs and epoxy resin.

### 3.2. The morphology of the composites

**Fig. 2:**
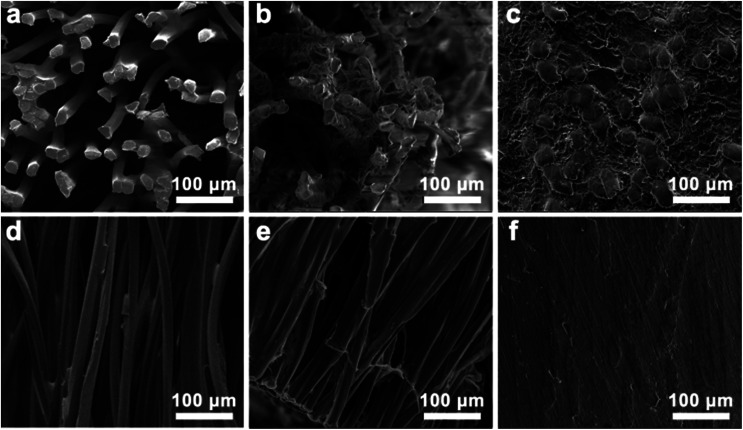
(a–f) SEM images of the CFs, CFTG, and CFTG/Ep composite. (a–c) Top-view of the CFs, CFTG, and CFTG/Ep composite. (d–f) Cross-sectional view of the CFs, CFTG, and CFTG/Ep composite.

Fig. 2 indicates the morphologies of the CFs, CFTG, and CFTG/Ep composite. Top- and cross-sectional views of the CF are shown in Fig. 2(a) and (d), respectively. The cigarette fibers were aligned with slight cross-linking, which was suggested to be beneficial for the integral strength of the CFs. The almost parallel cigarette fibers are considered essential foundation of the anisotropic structure and properties of the CFTG/Ep composite. The CFTG was obtained with GNPs attached to the surface of the CFs after its assembly.

Like CF, the CFTG also exhibited a typical anisotropic structure with a high aspect ratio, as can be seen in Fig. 2(b) and (e). This specific structure will lead to significant anisotropic thermal spreading properties. Fig. 2(b) revealed that the GNPs were wrapped closely on the surface of the CFs; this could explain the high thermal diffusivity observed in the vertical direction. Moreover, as shown in Fig. 2(b) and (e), only a thin layer of GNPs was attached to the CFs after 5 filtration cycles; this also indicated that the graphene loading in the CFTG/Ep composite was quite low. The wrinkled morphology observed in the top-view of the CFTG revealed that the CFs were partially connected together by the GNPs. However, the GNPs did not construct a continuous thermal transport network; thus, the thermal diffusivity of the CFTG/Ep composite in radial direction was improved slightly. The CFTGs remained in their original orientation in the epoxy matrix, as shown in Fig. 2(f), and the cross-sectional image of the CFTG/Ep composite exhibited isolated island-like fibers surrounded by the GNPs, which could further confirm the anisotropic filler structure. Fig. 2(c) and (f) also revealed that the anisotropic structure was not damaged after infiltration and curing of the epoxy resin. The unbroken anisotropic filler structure was the key factor of the improved and anisotropic thermal diffusivity of CFTG/Ep composite. As can be seen in Fig. 2(c) and (f), there were no air bubbles left; this meant that the vacuum treatment was very effective in eliminating the air bubbles during the curing process.

### 3.3. The thermal properties of the composites

**Fig. 3:**
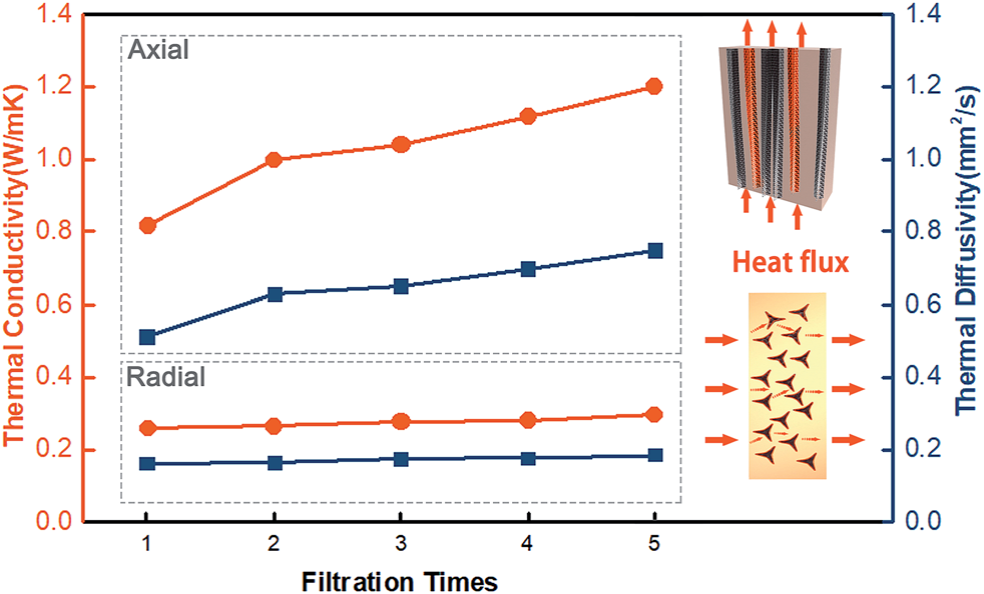
The thermal diffusivity and thermal conductivity as a function of the number of filtration cycles; the model of heat flow for the CFTG/Ep composites in the axial and radial directions is shown in the inset.

Fig. 3 reveals the thermal conductivity of the CFTG/Ep composite with a different number of graphene filtration cycles in the radial (⊥) and axial (∥) direction. It can be seen from Fig. 3 that the thermal conductivity of the CFTG/Ep composite is enhanced with an increase in the number of graphene filtration cycles. With one filtration cycle, the thermal conductivity of CFTG/Ep in the axial direction reached 0.816 W mK^−1^, which was about 3 times higher than that of pure epoxy. In addition, the thermal conductivity in the radial direction was only 0.261 W mK^−1^, and only slightly enhanced. This anisotropic thermal conductivity can be ascribed to the anisotropic structure of the CF. After one filtration cycle, the GNPs could be coated on the surface of CF and construct an effective heat conductive pathway in the axial direction; however, the GNPs could not build a continuous graphene network between the different CFs in the axial direction. Continuous GNPs networks can form effective heat conductive pathways and greatly improve the thermal conductivity of a composite. After five filtration cycles, the thermal conductivity of the composite in the axial direction increased up to 1.2 W mK^−1^; however, the thermal conductivity in the radial direction only reached 0.298 W mK^−1^. With an increase in the number of filtration cycles, more GNPs were coated on the surface of the CF, but the low thermal conductivity in the radial direction indicated that the GNPs did not build a continuous GNPs thermal conductive pathway in the radial direction. In addition, epoxy between the discontinuous graphene networks will also result in more phonon scattering and influence the thermal conductivity in the radial direction. In the axial direction, heat can be effectively transferred through the continuous graphene networks. The improvement in the thermal conductivity in the axial direction upon increasing the number of filtration cycles can also be attributed to the increase in the content of the GNPs.

**Fig. 4:**
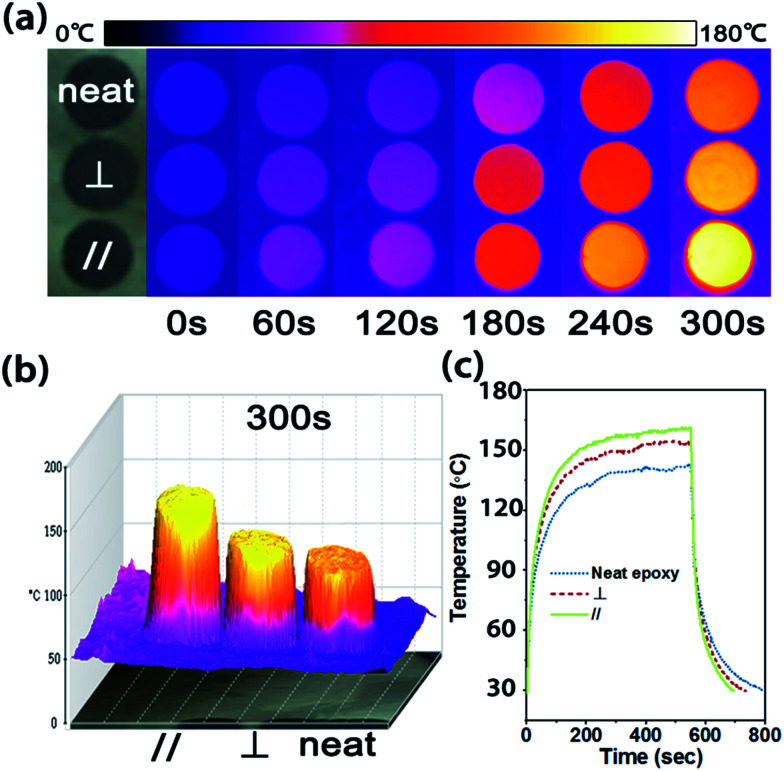
(a) The infrared images of neat epoxy and CFTG/Ep composite in the radial and axial direction upon heating. The temperature gradient scale bar on the top shows the highest and lowest temperatures of 180 °C and 0 °C, respectively. (b) The 3D infrared images of the neat epoxy and CFTG/Ep composite in the radial and axial direction after 300 s, (c) the surface temperature variation of the neat epoxy and CFTG/Ep composite in the radial and axial direction with time upon heating and cooling.

To visually verify the enhanced and anisotropic heat dissipation performance of the CFTG/Ep composite, we used an infrared camera to determine the variation in temperature in the composite and neat epoxy, as shown in Fig. 4. Initially, samples with the same size were prepared and carbon sprayed uniformly on their surface to eliminate the differences in the infrared emittance.

As shown in Fig. 4(a), the surface color of each sample becomes more and more bright in the infrared images as the temperature increases. After 300 s, the surface temperature of the CFTG/Ep composite in the axial and radial direction reached 155 °C and 140 °C, respectively, whereas the temperature of the neat epoxy was only 123 °C. The CFTG/Ep composite in the axial direction revealed a faster heating rate than that in the radial direction, which was in agreement with its anisotropic thermal diffusivity. The temperature difference was also consistent with the color of the different samples, which was clearly exhibited in the corresponding 3D infrared images, as can be seen in Fig. 4(b). The quantitative temperature profile evolution with time observed for the samples was determined using a multimeter under the same conditions, as shown in Fig. 4(c). The samples were cooled from 540 s, and the CFTG/Ep composite in the axial direction reached room temperature first. It can be easily seen that both the heating rate and the cooling rate of the composites in the axial direction are the highest. This result demonstrates that both the axial and radial direction of the CFTG/Ep composite exhibit an enhanced thermal diffusion ability than neat epoxy. Our experiences have investigated the application of the CFTG/Ep composite, and the composites in the axial direction as compared to neat epoxy and the composite in the radial direction have been indicated to be more effective for thermal dissipation.

## 4. Conclusions

A CFTG/Ep composite with significant anisotropic thermal diffusivity was fabricated using a facile filtration method. The thermal conductivity of the composite in the axial direction was 1.2 W mK^−1^ after 5 filtration cycles, whereas the thermal conductivity in the radial direction was only 0.298 W mK^−1^. The thermal conductive anisotropy can be ascribed to the anisotropic structure of the cigarette fiber-templated graphene. The excellent thermal transportation properties allow the epoxy composites to be used as an efficient heat dissipation material for thermal management applications.

## Conflicts of interest

There are no conflicts to declare.

## Supplementary Material
